# Identifying quality of life indicators to improve outpatient pharmacy services for prostate cancer patients: a comparison between brazilian and british experiences

**DOI:** 10.1590/S1677-5538.IBJU.2018.0553

**Published:** 2019-07-27

**Authors:** Harindra Patel, Patrícia Melo Aguiar, Adalberto Pessoa, Sílvia Storpirtis, Paul F. Long

**Affiliations:** 1 School of Cancer & Pharmaceutical Sciences, King's College London, United Kingdom, UK; 2 Departamento de Farmácia. Faculdade de Ciências Farmacêuticas da Universidade de São Paulo - USP, São Paulo, SP, Brasil; 3 Farmácia Universitária da Universidade de São Paulo - USP (FARMUSP), São Paulo, SP, Brasil; 4 Departamento de Tecnologia Bioquímico-Farmacêutica, Faculdade de Ciências Farmacêuticas da Universidade de São Paulo - USP, São Paulo, SP, Brasil

**Keywords:** Prostate, Neoplasms, Quality of Life

## Abstract

**Objectives:**

Prostate cancer is the most common and fatal cancer amongst Brazilian males. The quality of prostate cancer care in Brazil was systematically reviewed and compared to United Kingdom (UK) National Institute for Health and Care Excellence (NICE) guidelines, which are considered an international benchmark in care, to deter- mine any treatment gaps in Brazilian practice.

**Materials and Methods:**

A systematic review of Brazilian and UK literature was under- taken. Additionally, quality of life scores was measured using a FACT-P questionnaire of 36 prostate cancer patients attending the Farmácia Universitária da Universidade de São Paulo (FARMUSP). These scores were compared against NICE care measures for patient safety, clinical efficacy and quality of life indicators determined by either quantitative or qualitative methods.

**Key findings:**

The quality of prostate cancer care in Brazil was considered good when compared to NICE guidelines. However, FACT-P data strongly indicated a poor under- standing of treatment received by Brazilian patients and that their mental health needs were not being met.

**Conclusions:**

NICE quality statements that address the holistic needs of patients should be implemented into Brazilian outpatient care plans. Addressing the non-medical concerns of patients may improve quality of life and can be easily rolled-out through existing Brazilian pharmacy services at no financial cost to the Brazilian Unified Health System (SUS).

## INTRODUCTION

Survival rates amongst prostate cancer patients receiving treatment from the public healthcare system in Brazil (Unified Health System-SUS) are poor (1). Currently, treatment is based on the European urology guidelines which considers quality of life, but does not include outpatient pharmacy provision. Prostate cancer is the second most prevalent cancer in men worldwide, accounting for over one million diagnoses in 2012, equivalent to 1 in 7 of all new cancers diagnosed. In Brazil, there were approximately 75.000 new cases in 2017, more than any other cancer (2). The rise in prevalence is thought to be due to increase in life expectancy and improved diagnostic screening services in Brazil. Approximately 1.8% of men aged 40-60 have prostate cancer with the incidence increasing dramatically to over 14% for men aged 60-80 (3). The incidence of prostate cancer is different between ethnicities. Brazil's latest census in 2010 suggested over 80 million people was of Black African origin, approximately 42% of the population at the time, a 6% rise from 2000 (4). Black men are almost twice as likely to be diagnosed with prostate cancer in their lifetime, with higher pre-clinical disease and malignancy rates compared to Brazilian Caucasians (5, 6). Brazil has followed a global trend and improved quality of prostate cancer treatment in parallel with the country's socioeconomic development (7, 8). Current treatment options include surgery, chemotherapy, androgen deprivation therapy and radiotherapy (including brachytherapy). Each of these treatments can have adverse effects such as a decrease in libido, erectile dysfunction and osteoporosis which can have a psychological impact on patients causing them to experience anxiety and depression during treatment (9, 10).

In the United Kingdom, healthcare is benchmarked against guidelines issued and frequently reviewed by the National Institute of Clinical Excellence (NICE) (11). Three NICE measures of care are patient safety, clinical effectiveness and quality of life. Quality of life is measured subjectively and can be further broken down into patient satisfaction, health and happiness. NICE guidelines are based on evidence collected through stringent testing and developed by independent healthcare experts. The guidelines are important as a benchmark as they aim to ensure all patients receive the best possible care, whilst remaining cost-effective. NICE compare interventions against quality adjusted life years (QALY) and interventions exceeding £20000/QALY are not considered cost-effective.

Farmácia Universitária da USP (FARMUSP) (University Pharmacy of Sao Paulo State University) is an education and public outreach activity run by the Department of Pharmacy, Faculty of Pharmaceutical Sciences, University of São Paulo. Part of the outreach activities includes a study of prostate cancer patients aged 60+ with the objective to improve care in line with objectives outlined by the Brazilian Unified Health system (SUS) to incorporate outpatient pharmacy intervention following cancer treatment. In this study, patients attend monthly consultations with pharmacists at FARMUSP to monitor their overall well-being. The pharmacists promote healthy lifestyles through interventions and provide patients with information about their on-going treatment (12, 13). Herein we systematically review the literature on prostate cancer care in Brazil and in the United Kingdom, as well as incorporating data collected by FARMUSP that measures quality of life, patient safety and clinical effectiveness. We aim to identify whether elements of NICE guidance can be used to improve the management of prostate cancer patients post-treatment, in the Brazilian public healthcare system.

## MATERIALS AND METHODS

### Study design

A PRISMA checklist and flow diagram were followed to ensure the quality of the literature search was equivalent to other systematic reviews (14). Searches were made using the PICO search strategy protocol, which helped define the search question (15). In addition to the systematic review, data collected by the pharmacists at FARMUSP on male prostate cancer patients was additionally analysed. These data were collected from October 2014-present, in the form of monthly or annual questionnaires, where patients were treated with either cyproterone acetate, goserelin or both.

### Framework of the question

The PICO elements were modified according to the nature of the question, which is based on therapy/treatment (15). Table-1 shows the breakdown of the altered PICO framework used to search in this study.

**Table 1:** PICO framework of study inclusion.

Question type	Patient Problem	Intervention	Comparison	Outcome measures
Therapy	Prostate cancer patients in Brazil	Pharmaceutical care-i.e. if pharmaceutical management has improved quality of life in Brazil	Care standards in Brazil and the UK	Improvements in: Clinical outcomes – guideline efficacy Quality of life - questionnaires (FACT-P HADS), structured interviews Patient safety - adherence, mortality rate

### Study criteria

There were no limits of patient ethnicity, age, drug treatment or other medical conditions. Only articles published after 2012 were used to maintain relevance to the most current standards of care. Published articles, which were not in English, were excluded due to a lack of translation facilities. The preferred study designs were higher up the hierarchy of evidence. The criteria for studies are shown in Table-2.

**Table 2:** Inclusion and exclusion criteria of study.

Question component	Inclusion criteria	Exclusion criteria
Population	Male prostate cancer patients/ professionals in Brazil and UK who have/ manage prostate cancer	Other patients, health care professionals or countries
Interventions	The quality of pharmaceutical care in Brazil and the UK with NICE guidance	Lack of pharmaceutical care measure
Outcomes	Clinical, patient safety - adherence, quality of life – health, happiness, satisfaction	Unrelated outcomes
Study design	Randomised control trials, cohort studies, systematic reviews, clinical guidelines	Other study designs

### Overview of the search strategy

The keywords and concepts for the search were developed from the PICO framework. When searching the EMBASE database, MeSH (Medical Subject Headings) terms were used to focus the search. The MeSH terms used, based on NICE guidance measure of care were: prostate cancer/patient safety/clinical effect/quality of life/mortality. A complete search in the Ovid Embase database is shown in Table-3. Additionally, this strategy was used to search the Cochrane library, Medline and PubMed databases with varying limits according to availability. The bibliographies of relevant articles were also searched, as well as the following journals: Journal of Research in Pharmacy Practice; European Journal of Hospital Pharmacy; Pharmaceutical Journal; British Medical Journal; European Journal of Urology and the International Brazilian Journal of Urology.

**Table 3:** Ovid Embase database search terms.

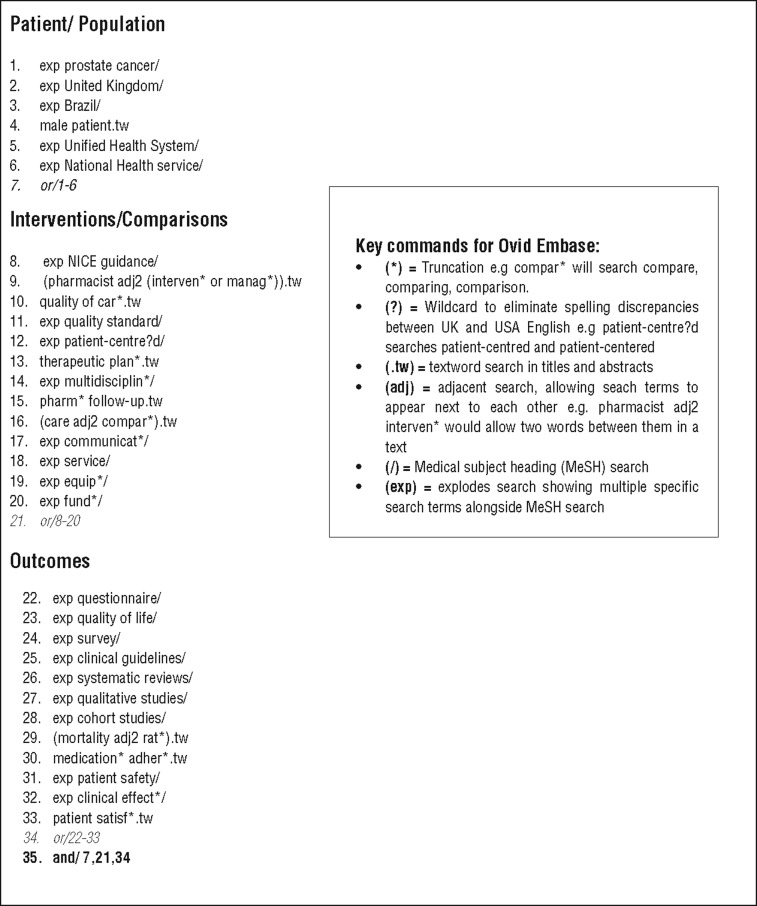

### Step-by-step electronic search used on Ovid Embase

#### Selection of studies

All searches were completed and all articles were imported into Endnote and duplicate articles were removed. The titles of articles were read and evaluated for relevance, followed by abstracts. Potential articles were then read in full and were measured against the inclusion and exclusion criteria to determine eligibility.

### Data collection process

Data was extracted from studies that met the inclusion criteria through a customised extraction form in Microsoft Excel. The variables of the form were:

- Study title/design
- Origin country of study
- Gender/Age/Number of participants in study
- Duration of study
- Measure of adherence/patient safety? OR
- Measure related to quality of life? OR
- Measure of clinical outcomes?
- Tools of measure i.e. questionnaires
- Decision of inclusion based on form completion-with a justification
- Assessment of bias risk and quality

Studies that met the inclusion criteria were allocated a quality assessment ranking and examined for potential bias based on questions from the critical appraisal skills programme (CASP) (16). CASP lists help assess validity, clinical importance and relevance of studies to reduce bias through a series of closed questions. CASP was appropriate in this study as multiple lists were available according to different study designs. The questions for systematic reviews assessed: focus of question; importance of study; precision of results and whether the results could be applied to the local population. For qualitative studies: clear aims; appropriate methods; recruitment strategy; ethical considerations; rigorous data analysis and value of research were assessed. Cohort studies were assessed by: appropriate population; subjective/objective measures to reduce bias; appropriate measure of outcome; consideration of confounding variables; appropriate length of study and precision of results. The generated scoring system used for assessment of quality based on CASP, to enable comparison, is shown in Table-4. The mean score for each study was calculated and an overall quality ranking was determined as shown in Table-5.

**Table 4:** The scoring system for individual questions in the CASP list.

Answer to closed question	Score allocated
Yes	3
Can't tell	2
No	1

**Table 5:** Overall quality ranking allocation of each study based on mean CASP score list.

Mean CASP list score	Quality assessment ranking
≤ 1.4	Low
1.5-2.4	Medium
≥2.5	High

### Summary measures

Studies were individually reported and summarised with measures of quality of life, clinical effectiveness and patient safety. Where appropriate, the differences between the three measures were reported.

Quality of life measure for FARMUSP recruited patients Each patient completed the FACT-P (version 4) questionnaire (www.facit.org/LiteratureRetrieve.aspx?ID=42292) which assessed quality of life through a series of closed questions. The questionnaire was made available in Portuguese to eliminate bias (17). This questionnaire was completed before drug treatment and annually thereafter. A score was calculated as the sum of the following subscale scores within the questionnaire: Physical well-being (PWB-7 items) + Functional well-being (FBW-7 items) + Emotional well-being (EWB-5 items) + Social well-being (SWB-7 items) + Prostate cancer subscale (PCS-12 items). Answers ranged from ‘not at all’ (score of 0) to ‘very much’ (score of 5). Higher scores indicate a higher quality of life, with scoring being reversed on negative-based questions. Only questionnaires with a minimum of 80% response rate were considered appropriate (18). The median FACT-P scores, along with median of the various subscales, were recorded for patients before drug treatment at FARMUSP, one year after and two years after to measure whether quality of life had changed.

## RESULTS

### Study selection

**Figure 1:**
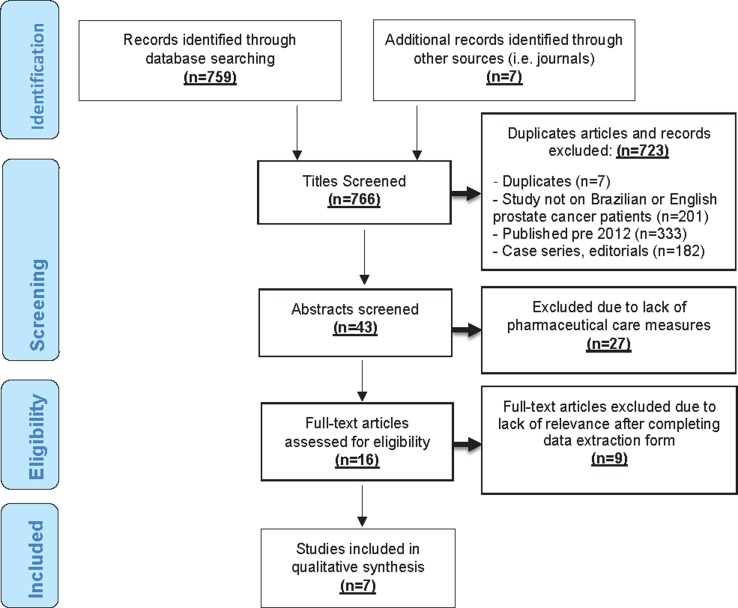
PRISMA flow diagram showing the results of the literature search.

The PRISMA flow diagram shown in Figure-1 gives the results of the literature search. Some 766 articles were identified through database and online journal searches. Of these 723/766 (94%) of the articles were excluded after title screening, including 55 duplicated records. The remainder of the excluded articles wither had no relevance to prostate cancer, were published pre-2012, were studies outside of Brazil or the United Kingdom, or did not meet the correct study design. Of the 43 abstracts that were read, 27 articles were further excluded due to a lack of clear pharmaceutical care measures and outcome relevance. After full text searches, 7 articles were selected for qualitative synthesis, after completion of the data extraction form to assess relevance.

### Study characteristics

Seven articles complied with the complete inclusion criteria. The main concepts and findings are summarised in Table-6. Studies were published between 2012 and 2017 and all studies were conducted in either the Brazil or the United Kingdom. Various study designs were used including a retrospective cohort study (1/7), cross-sectional studies (3/7), a clinical consensus study or questionnaire (2/7) and a literature review (1/7). The studies measured quality of care through different measures. Braga (1) used data on 16.280 prostate cancer patients from the Brazilian Base Oncology database to evaluate risk of death 5 years after diagnosis as a measure of patient safety. Sasse et al. (19), interviewed an 18-man panel of prostate cancer specialists to evaluate their views on clinical effectiveness of the Brazilian prostate cancer guidelines. Nardi (20), used information on patients from 1082 physicians from the Brazilian Urology Society to evaluate the differences in clinical effectiveness between public and private health care systems in Brazil. Paterson (21), interviewed 31 prostate cancer patients with ≥ T3 staging in the United Kingdom and measured physical, psychological and social quality of life, to provide information to improve patient-centred care. Paterson (21), measured quality of life through literature review of NICE prostate cancer guidance, to ensure information on diagnosis and management was made clear for healthcare providers. Watts et al. (23), used cross-sectional questionnaires to assess quality of life via measurements of clinical anxiety or depression in 313 prostate cancer patients under active surveillance in the UK. Payne (24), questioned 61 oncologists working in the United Kingdom public health system to evaluate their opinions on the clinical effectiveness of the NICE guidance for prostate cancer treatment.

**Table 6:** Summary of selected studies measuring prostate cancer care in Brazil and the UK.

Study design and author	Population/Information studied	Type of care quality measured	Method of measurement	Core outcomes
Braga (1) Retrospective cohort study Brazil study	16280 prostate cancer patients (staged I-IV) treated in the Brazilian Unified Health system between 2000-2006	The mortality rate of prostate cancer patients	Patient information from Base Onco was used to predict overall survival after 5 years through application of the Kaplan-Meier method. Prostate cancer specific survival was predicted by applying Fine and Gray's competitive risks model	- Approximately 25%(n=3160) of patients died due to prostate cancer* -Probability of overall survival = 0.5* - Probability of specific survival = 0.7* *= (after a minimum of 5 years) - Increased mortality due to late diagnosis, poor cancer treatment and declining medical conditions
Sasse et al. (19) Clinical consensus Brazil study	18-man panel of professionals in the field of prostate cancer from Brazil. Made up of oncologists, urologists and radiooncologists	Clinical effectiveness of treatment via the Brazilian prostate cancer guidelines	An adapted model of the St. Gallen Advanced prostate cancer consensus conference was used to generate 40 questions on epidemiology, treatment of local prostate cancer and screening. The specialists had 2 months to analyse. Each question was based on current guidelines, needed 2/3 of the panel vote for consensus to potentially change	- Consensus on keeping serum testosterone below 50 ng/dL for castration - Consensus 71%(n =13) agreed intermittent hormonal blockage is appropriated in specific patients - Consensus 100% (n=18) that rise in PSA whilst on androgenic suppression defines castration resistance - Many areas showed no clear consensus, indicates weak evidence available
Nardi (20) Cross-sectional web based survey Brazil study	1082 physicians from Brazilian Urology Society providing data on their prostate cancer patients	Clinical effectiveness of different treatments, comparing public and private health care	Questionnaire emailed to urologists regarding information on clinical, pathological features (Gleason score) of prostate cancer as well as socioeconomic factors. TNM staging was also measured. Clinical data was analysed descriptively whilst the chi-square test compared the amount of variation between groups	- Median PSA value = 10 ng/mL - Most frequent Gleason score was 5-6 52% patients (n= 531) - In public system a prostatectomy was most common initial care 47% patients (n=485) - Higher median PSA in public system than private (10 vs 6.8 ng/mL; P<0.001) - More patients in private system with no health insurance had to move for treatment than when treated publically (73% n= 126 vs 69% n= 705; P<0.0001)
Paterson (21) Mixed methods study Cross sectional studies Semi-structured interviews UK study	31 men with ≥ T3 stage prostate cancer from the UK	Quality of life measure through physical, emotional sexual well being	The supportive care needs survey was completed by patients to measure physical living, health, physiological needs and patient care. The self-efficacy scale was completed to measure their self-management. The European organisation for research and treatment of cancer quality of life of prostate cancer was completed to assess quality of life. Questionnaires were analysed using SPSS.	- Reduced level of selfefficacy was reported in comparison with literature - 42% (n=13) reported lack of supportive care and empathy in relation to information of cancer spread -33% (n=10) reported increased fatigue - 30% (n=9) reported felt results were out of their control -30% (n=9)felt they were not given adequate information - Men reported a lack of overall understanding of their treatment
Paterson (22) Literature review UK study	NICE guidelines on prostate cancer treatment	Quality of life measure through ensuring guidance is clear on how prostate cancer is diagnosed, progresses, managed and provides information for patient education	Literature review conducted across electronic databases, searching quantitative and qualitative studies. UK and European guidelines also reviewed. Guidelines and article information on diagnosis, management were narratively assessed	- Multidisciplinary team important to provide consistent high quality treatment - The use of patient reported outcomes (PROMS) are essential to overcome unmet supportive care needs - A holistic needs assessment (HNA) helps tailor and improve care for individual
Watts et al. (23) Cross-sectional questionnaire survey UK study	313 men diagnosed with prostate cancer who were managed by active surveillance across urology departments from the UK	Quality of life measure, through prevalence of clinically meaningful anxiety/depression after prostate cancer diagnosis	Selected patients completed a hospital anxiety and depression scale questionnaire (HADS). Patients with a score of 8 or more were considered to have depression/anxiety. Social and demographic information was also obtained via questionnaire, with only data with a P<0.05 considered significant	- Depression scale showed 13% (n=39) of patients had a score of ≥8 = clinical depression - Anxiety scale showed 23% (n=73) had a score of ≥8 = clinical anxiety - Only one demographic, Southampton, showed statistically significant probability (P<0.0005) of increased depression in men
Payne (24) Semi-structured questionnaires UK study	61 oncologists from the NHS	Evaluating clinical effect of treatment via NICE guidance pre- 2014	72 question survey completed initially (2008). Followed by a 2nd focused questionnaire (2010) with 22 questions assessing adherence to clinical guidelines and whether practice had changed after 2 years	- 60% of participants felt NICE guidance would improve prostate cancer care - 30% of participants felt NICE guidance would degrade treatment - 61% of participants felt NICE guidance required updating

### Effects of interventions on clinical effectiveness

Sasse et al. (19), aimed to gain consensus of various clinical guidelines through a 2/3 vote of an 18-man specialist panel. A review of the literature concluded that 67% (n=12) believed additional treatment was unnecessary in undetectable metastasis from imaging due to a lack of evidence. The majority of the literature, 88% (n=16) considered the first line use of abiraterone/enzalutamide. Nardi (20), showed surgery was recommended most frequently as a first line treatment in the Brazilian public system (47% n = 485), followed by radiotherapy with/without hormone ablation (27% n = 278) and orchiectomy (20% n = 208). Patients receiving treatment from the public healthcare system had a greater median PSA (10 vs. 6.8ng/mL; P < 0.001) with a higher probability of being diagnosed with metastatic disease (10% n = 103 versus 4% n = 35; P < 0.001) than patients treated privately. Payne (24), found that 60% (n = 37) of British oncologists in 2008 felt NICE guidance would improve patient care. There was split, with 49% (n = 30) voting against active surveillance as first line treatment for low risk localised prostate cancer. Only 56% (n = 34) believed follow-up appointments for men with a stable prostate-specific antigen (PSA) for ≥ 2 years were essential. Results from 2010 show a rise in favour of active surveillance with 80% (n = 62) agreement. There was a slight increase in favour of primary care follow-up to 59% (n = 450). These findings reiterate the need for constant updating of NICE guidelines to improve clinical outcomes.

### Effects on interventions on quality of life

Sasse et al. (19), showed a vote for the introduction of intermittent hormonal blockage (71% n = 13) for asymptomatic patients with confirmed metastasis in Brazil. This was based on studies that permanent hormone suppression may lead to increased side effects and a decreased quality of life (25). Nardi (20), showed treatment in the Brazilian public health system resulted in almost 69% (n = 705) of patients having to leave their home city for treatment on a regular basis. Paterson (21), showed 84% (n = 26) of men with prostate cancer in the UK were fatigued, 57% (n = 17) exhibited insomnia and, almost 50% (n = 15) were dissatisfied with their sex life. Of the 8 participants who completed in depth interviews: (1/8) complained of lack of sympathy when informed about his cancer spreading, (2/8) emphasised they required emotional support, (1/8) complained of extreme back pain, (1/8) about mobility issues due to urinary incontinence and (3/8) stated that a better communication with their healthcare team would help optimize care and improve life quality. Paterson (22), through review of the literature concluded multidisciplinary teams must work together with patients in the UK to enhance personalised care and follow evidence-based approaches to tailor treatment. The need for patient reported outcomes (PROMS) was addressed to help understand patients desired outcomes and provide treatment accordingly. The use of a holistic needs assessment (HNA) was found to help specialists provide treatment, taking all factors into account including patient needs. Watts (23), found after completion of Hospital anxiety and depression score (HADS) in United Kingdom prostate cancer patients that the mean HADS-depression score was 3.3, with 13% (n = 39) of patients having a score of ≥ 8 = clinical depression. The mean HADS-anxiety score was 4.8, with 23% (n = 73) of patients having a score of ≥ 8 = clinical anxiety. From these results, a need for new strategies to improve patient management and quality of life was clearly identified in United Kingdom patients receiving treatment for prostate cancer.

### Effects of interventions on patient safety

Braga (1), found there was a mean time of 5 months between diagnosis and outpatient cancer treatment (SD of 6 months). On average, patients were followed up for 51 months (SD of 26 months). Patient survival rate was 0.5 after 5 years. This decreased with age and a higher cancer staging with a 0.65 survival probability in stage I compared to 0.35 probability in stage IV (after 5 years). Hospitalisation in the SUS system increased the risk of death by 67% after 5 years. Sasse et al. (19), showed a vote against the indication of ciproterone acetate (79% n = 14), although widely prescribed as a front-line treatment for prostate cancer in Brazil, based on studies indicating a decrease in patient survival rate (26).

### Quality and bias risk assessment

The critical appraisal skills programme (CASP) listed scores and quality assessment rankings for all the studies recovered from the search and are given in Table-7, with a summary. All studies had problems with methodology, for example the studies of Paterson (21) and Watts et al. (23), both contained mixed methods, which meant that any complete comparison between studies would not be possible.

**Table 7:** Quality assessment of selected prostate cancer studies using CASP tools.

Study	Mean CASP list score	Quality summary	Quality assessment
Braga (1)	2.5	16820 prostate cancer patients were analysed- a large sample. Cohort studies are higher on the hierarchy of evidence. However, not enough factors were considered when estimating risk of death.	High
Sasse et al. (19)	1.4	Only 18 panellists. Voting was subjective and based on varying level of experience of the Prostate cancer specialists, which was not stated.	Low
Nardi (20)	2.4	Large sample size, with many factors considered and unbiased statistical analysis with use of chi-square test. However, some data was measured subjectively with lack of evidence.	Medium
Paterson (21)	1.8	Small study with only 8 patients completing full interviews, however they were described in sufficient detail. Closed question survey for 31 patients enabled in-depth quantitative analysis.	Medium
Paterson (22)	2.4	Research question defined with clear results, which can be applied to the population. However, not all outcomes were considered – lack of clinical evidence.	Medium
Watts et al. (23)	1.6	Reasonably sized sample size of 313 patients, with good statistical analysis of data. However, not enough factors considered and demographic data lacked validity as only one result met the specified significance value of P<0.05.	Medium
Payne (24)	1.3	There was no information on the background of the 61 oncologists questioned, including their level of experience. Answers were closed and there was no reasoning given for answers. Only subjective measures were used.	Low

**CASP** = critical appraisal skills programme; CASP score of ≤1.4 = low quality, 1.5-2.4 = medium quality, ≥ 2.5 = high quality.

### FARMUSP patient quality of life results

Before treatment, 34 patients completed the FACT-P questionnaire. Only 13 patients had completed a questionnaire 2 years into their treatment and these data are given in Table-8. The number of patients at each stage of the assessment decreased as patients joined the programme after 2014, so only completed their initial questionnaires. Some patients died during treatment. Mean FACT-P scores decreases from 122 (before treatment) to 119 (2 years into treatment). Scores corresponding to the responses to individual questions consistently gave large and overlapping standard deviations. From a quantitative perspective these large standard deviations might suggest little difference in the comparative scores, but from a qualitative perspective indicated that patients felt differently about their quality of life during treatment.

**Table 8:** The average (median) FACT-P scores of patients before and after treatment.

	Median score before treatment (n=34)	Median score 1 year into treatment (n=23)	Median score 2 years into treatment (n=13)
FACT - P	122 (19)	120 (21)	119 (21)
PWB	24 (4)	23 (4)	25 (4)
SWB	20 (4)	21 (4)	20 (5)
EWB	21 (4)	19 (4)	19 (4)
FWB	20 (5)	19 (5)	19 (6)
PCS	36 (6)	36 (8)	37 (6)

**PWB** = physical well-being; **SWB** = social well-being; **EWB** = emotional well-being; **FWB** = functional well-being; **PCS** = prostate cancer subscale; **A** = higher score indicates a better quality of life; (19) indicates a standard deviation of 19.

## DISCUSSION

This study systematically reviewed the literature on the quality of care of patients during and after treatment for prostate cancer in Brazil, in comparison to patient experiences and NICE guidance in the United Kingdom. In addition to the systematic review, data were also collected by the pharmacists at FARMUSP, an outreach pharmacy service currently piloting a survey to measure quality of life, patient safety and clinical effectiveness in prostate cancer patients attending the University of São Paulo Hospital. Inclusion of data from this cohort of 36 patients was necessary because of a paucity of quality of life and patient safety adherence literature on Brazilian prostate cancer patients. Quality of life and patient safety adherence are two of the three elements of NICE guidance that measure of care quality (the other being clinical efficacy). These two elements are examples of patient-centred care, which is considered the root of best practice when assessing the overall quality of patient care (12, 13). Our combined systematic review and patient cohort data would suggest that current treatment plans in Brazil fall short of NICE guidance with respect to quality of life measures, especially mental health and treatment information provisions. Specifically addressing these patient needs may become increasingly apparent if the prevalence of prostate cancer continues to rise in Brazil.

The study process had many limitations at review and outcome level. The search strategy was comprehensive but only completed by one person. This may have led to incorrect omission of reports, as there was no verification when assessing reports against exclusion and inclusion criteria. Secondly, some case reports from Brazil were not included, as these were not in English. There was also a time constraint, which meant not all relevant databases or articles could be searched during the project lifetime. At outcome level, the studies were heterogeneous and due to the differences in care measure, it was not possible to statistically combine the results between studies. Only the data reported by Braga (1) had a high quality assessment ranking, whilst the remainder of the studies were ranked medium to low quality. The lower quality ranking assigned to the studies of Sasse et al. (19), Paterson (21) and Payne (24) was probably due to the use of selective measures of care and low sample numbers, which increased the risk of bias. The data reported by Watts et al.(23) could not be adequately assessed, as the outcome being measured was not clear. There was minimal consideration of confounding variables throughout all of the studies, which reduced the objectivity of the results. The measure of quality using CASP was partially subjective and only based on a single opinion, which reduced the accuracy of quality rankings. The data collected at FARMUSP also had limitations. Quality of life data were collected at the midpoint of a five-year trial; therefore, it is difficult to project a measure of the level of long-term care. The FACT-P questionnaire measured overall quality of life, which may have been affected by external factors not related to prostate cancer.

Braga (1) identified that almost a quarter of patients (n = 3160) died within 5 years of diagnosis when treated in the Brazilian public healthcare system. In contrast, 84% of prostate cancer patients survived after 10 years following UK public healthcare treatment (27). NICE measures care through patient safety and implements measures for prevention of premature death in prostate cancer patients (28). A quality standard measure used in NICE is to include evidence of local arrangements made to discuss treatment options with an oncology specialist. This could be implemented into the Brazilian system to ensure patients of all backgrounds have access to local care which would improve patient safety. Sasse et al. (19), generally suggests specialists are in agreement of treatment options to maximise clinical effect and improve patient safety, however identified a lack of evidence. This identified a need for national treatment guidelines in Brazil that parallel those of NICE that are more inclusive of the patient when making treatment decisions. In contrast, Payne (24) identified that many oncologists in the UK did not agree, or adhere with NICE guidance, as some patients required more individualised treatment specific to their cancer. This reiterates the need for constant review and updating of guidelines as the landscape of treatment options and patient expectations change. Nardi (20), suggested that almost half of the patients surveyed (n = 485) received radical prostatectomy as first line treatment. However, studies in the United Kingdom have shown active surveillance rather than surgery was just as effective a treatment option when patient quality of life and mortality were measured over 10 years following diagnosis (29). Watts et al. (23), measured depression and anxiety in prostate cancer patients in hospitals within the United Kingdom. Mental health was not addressed specifically in any of the Brazilian studies, and implementation of an anxiety/depression scale would, therefore, greatly aid identifying patients that may need further support to improve their quality of life.

Paterson 2017 (21) and Paterson 2015 (22), identified that in the United Kingdom, when a holistic needs assessment (HNA) was introduced into a patient care plan, communication between patient and healthcare teams was greatly improved. The HNA helps identify physical, practical, family and emotional concerns. A protocol to measure the unmet needs of prostate cancer patients was completely lacking in the literature we systematically reviewed from Brazilian studies. An HNA protocol had also not been implemented at FARMUSP and the effects of not considering the holistic needs of the patient were reflected by the FACT-P results, which showed a large variation in quality of life with no clear improvements. Implementing a HNA protocol under regularly reviews, could allow patients to communicate specific problems, not just medical related problems restricted to fixed questions from FACT-P. This can allow the healthcare professional, for example a pharmacist, to pinpoint potential problem areas which could then be addressed by the treatment team, thereby optimising care and improving quality of life. A HNA can be implemented with little or no financial expenditure, and the benefits of these interventions can be directly measured by inclusion into existing cost models of prostate cancer treatment in Brazil (30).

## CONCLUSIONS

This study has highlighted that current prostate cancer care plans in Brazil are of a high international standard, but important improvements can made because existing care does not consider the holistic needs of patient. Including a HNA protocol into patient care plans is an intervention that can be immediately implemented. The use of an anxiety/depression scale is also an intervention that can be rolled-out into the Brazilian Unified Health System (SUS)with immediate effect, to help establish if mental health is affecting prostate cancer patient quality of life. Both interventions can be made with little or no fiscal spend by the public healthcare system. A follow-up study, specifically measuring quality of life indicators should now be considered, to determine if these two simple interventions have a positive impact on clinical practice in Brazil.
